# Editorial: The Role of Neuropeptides in Sleep/Wakefulness States and the Circadian Clock

**DOI:** 10.3389/fnins.2022.913371

**Published:** 2022-05-09

**Authors:** Natsuko Tsujino, Masayuki Ikeda, Arisa Hirano, Takeshi Sakurai

**Affiliations:** ^1^International Institute for Integrative Sleep Medicine, University of Tsukuba, Tsukuba, Japan; ^2^Graduate School of Science and Engineering, University of Toyama, Toyama, Japan

**Keywords:** sleep, circadian rhyme, neuropeptide, metabolism, preoptic area of hypothalamus, arousal, SCN

Sleep is observed in most animals and is a crucial physiological function to maintain their lives. However, fundamental questions about sleep remain a mystery, such as why we sleep, what mechanisms regulate sleep, and what is happening in the sleeping brain?

In recent years, neuropeptides and/or neurons that produce neuropeptides have been shown to play important roles in the regulation of sleep and wakefulness, and research on these peptides has made breakthroughs in the understanding of sleep research.

This Research Topic includes two original papers and five review articles that demonstrate the multifaceted involvement of various neuropeptides in sleep and circadian rhythms, and we hope that this Research Topic will further advance sleep and circadian rhythm research.

Sleep and metabolism are known to be closely related. Makino et al. investigated how metabolism and feeding regulate sleep using satiety quiescence behavior as a readout in *Caenorhabditis elegans*. The authors performed an RNAi-based screening to identify neuropeptides that regulate satiety-induced sleep in *C. elegans*. Among 28 tested RF amide-related peptides genes, they identified FLP-11 in the RIS neuron as a major regulator of satiety quiescence. Among 28 tested insulin-like peptides genes, they also identified *ins-1* as a negative regulator for satiety quiescence. The authors propose a comprehensive model to explain how feeding affects quiescence via peptide signaling in *C. elegans*.

In mammals, sleep and body temperature are also known to be closely related. The preoptic area of the hypothalamus (POA) serves as an essential brain region for the regulation of sleep and body temperature. Understanding how these two behaviors are regulated within the hypothalamus requires molecular identification of the relevant circuits and mapping their local and brain-wide connectivity. Rothhaas and Chung reviewed the current understanding of the regulatory mechanisms of sleep and temperature, with a focus on the recently discovered POA neurons that regulate sleep and body temperature. In addition, the authors discuss important unresolved issues such as the anatomical and functional overlap between sleep- and temperature-regulating neurons, their pathways, and the role of various signaling molecules.

The POA, located in the most anterior part of the hypothalamus, is a brain region that has a complex structure consisting of different groups of neurons that control various functions and behaviors including sleep, parental behaviors, and sexual behavior. Tsuneoka and Funato survey the cell types that make up the mammalian POA and attempt to summarize their role in those behaviors. This is a valuable review article for specialists trying to elucidate the functions of the cell types in the POA region.

Orexin and MCH neurons are known to be involved in sleep/wake regulation. On the other hand, non-canonical roles have been revealed in recent years. Concetti and Burdakov reviewed these recent studies showing that both orexin and MCH neurons can rapidly change their firing when awake animals experience external stimuli, or during self-paced exploration of objects and places. The authors present sufficient evidence supporting their perspective that the sensory and motor control by these neurons could be distinct from their “arousal” effects, and they point out the need to develop a scientific definition of “arousal.”

How does the brain control sleep/wake states and maintain their stability? Sleep stages are thought to be controlled by the activity of specific neurons and glial cells in the brain. Since sleep-waking is generated by subcortical neuronal populations, it is important to monitor subcortical neuronal activity. Shiromani et al. reviewed the latest technology, miniscope, for calcium imaging of specific neurons. They observed that the activity of most GABA and MCH neurons in the lateral hypothalamus is heavily biased toward sleep. Such technology will help us clarify how specific neural circuits are involved in sleep-wake control at the cellular level.

A master circadian pacemaker located in the suprachiasmatic nucleus (SCN) in the hypothalamus regulates the circadian rhythm of physiological and behavioral activities in mammals. Individual neurons throughout the SCN are intrinsic but unstable circadian oscillators, whereas intercellular coupling in the SCN results in coherent and robust circadian oscillators. In this review, Ono et al. discuss some of the latest advances in understanding the roles of the vasoactive intestinal polypeptide (VIP) and arginine vasopressin (AVP) within the SCN in the generation of circadian rhythmicity.

Is the SCN the only locus of circadian control in sleep, or do these local brain clocks also contribute? To test the role of extra-SCN clocks, Maywood et al. created temporally chimeric mice in which circadian timekeeping was intact in all cells but with contrasting genetically specified periods in the SCN and local clocks. The authors found that the sleep of chimeric mice was fragmented. The authors also found that chimeric mice also displayed reduced recovery sleep in response to sleep deprivation. Furthermore, the authors showed that the mice exhibited robust loss in recognition memory. This shows that synchronization of the SCN clock and local brain clocks is essential for sleep regulation and memory consolidation.

## Author Contributions

NT, MI, AH, and TS contributed to the writing of the manuscript. All authors contributed to the article and approved the submitted version.

## Conflict of Interest

The authors declare that the research was conducted in the absence of any commercial or financial relationships that could be construed as a potential conflict of interest.

## Publisher's Note

All claims expressed in this article are solely those of the authors and do not necessarily represent those of their affiliated organizations, or those of the publisher, the editors and the reviewers. Any product that may be evaluated in this article, or claim that may be made by its manufacturer, is not guaranteed or endorsed by the publisher.

